# Mixed Reality for Cranial Neurosurgical Planning: A Single-Center Applicability Study With the First 107 Subsequent Holograms

**DOI:** 10.1227/ons.0000000000001033

**Published:** 2023-12-29

**Authors:** Elisa Colombo, Luca Regli, Giuseppe Esposito, Menno R. Germans, Jorn Fierstra, Carlo Serra, Martina Sebök, Tristan van Doormaal

**Affiliations:** * Department of Neurosurgery, Clinical Neuroscience Center, Universität Zürich, Universitätsspital Zürich, Zurich, Switzerland;; ‡ Department of Neurosurgery, Clinical Neuroscience Center, Universitätsspital Zürich, Zurich, Switzerland

**Keywords:** Augmented reality, Mixed reality, Neurosurgery, 3D visualization, Surgical planning

## Abstract

**BACKGROUND AND OBJECTIVES:**

Mixed reality (MxR) benefits neurosurgery by improving anatomic visualization, surgical planning and training. We aim to validate the usability of a dedicated certified system for this purpose.

**METHODS:**

All cases prepared with MxR in our center in 2022 were prospectively collected. Holographic rendering was achieved using an incorporated fully automatic algorithm in the MxR application, combined with contrast-based semiautomatic rendering and/or manual segmentation where necessary. Hologram segmentation times were documented. Visualization during surgical preparation (defined as the interval between finalized anesthesiological induction and sterile draping) was performed using MxR glasses and direct streaming to a side screen. Surgical preparation times were compared with a matched historical cohort of 2021. Modifications of the surgical approach after 3-dimensional (3D) visualization were noted. Usability was assessed by evaluating 7 neurosurgeons with more than 3 months of experience with the system using a Usefulness, Satisfaction and Ease of use (USE) questionnaire.

**RESULTS:**

One hundred-seven neurosurgical cases prepared with a 3D hologram were collected. Surgical indications were oncologic (63/107, 59%), cerebrovascular (27/107, 25%), and carotid endarterectomy (17/107, 16%). Mean hologram segmentation time was 39.4 ± 20.4 minutes. Average surgical preparation time was 48.0 ± 17.3 minutes for MxR cases vs 52 ± 17 minutes in the matched 2021 cohort without MxR (mean difference 4, 95% CI 1.7527-9.7527). Based on the 3D hologram, the surgical approach was modified in 3 cases. Good usability was found by 57% of the users.

**CONCLUSION:**

The perioperative use of 3D holograms improved direct anatomic visualization while not significantly increasing intraoperative surgical preparation time. Usability of the system was adequate. Further technological development is necessary to improve the automatic algorithms and reduce the preparation time by circumventing manual and semiautomatic segmentation. Future studies should focus on quantifying the potential benefits in teaching, training, and the impact on surgical and functional outcomes.

## Graphical abstract

**Figure:**
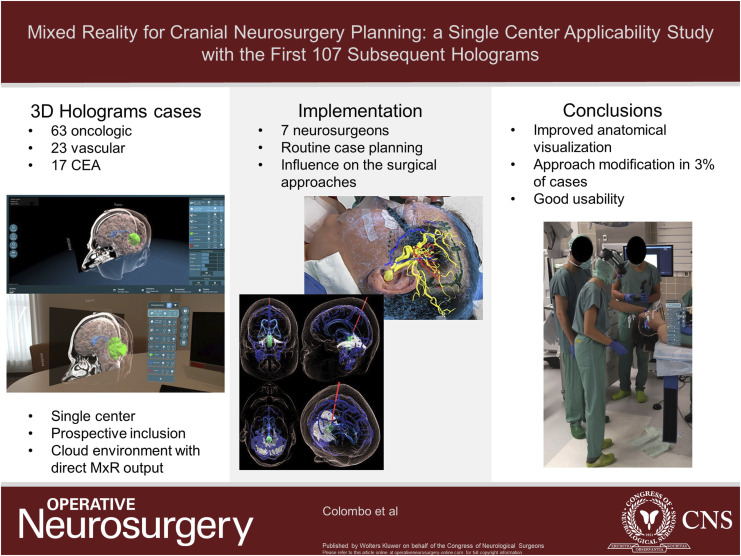

**ABBREVIATIONS:**
AR: augmented reality
MxR: mixed reality
NA: nonapplicable
USE: Usefulness, Satisfaction and Ease of use
VR: virtual reality.

With the superimposition of a computer-generated image on a user's view of the real world, augmented reality (AR) has been one of the major sources of 3-dimensional (3D) visualization and simulation. Among the different forms of AR, mixed reality (MxR) has been emerging in neurosurgery.^1^ In MxR, 3D objects called holograms recapitulate the patient-specific cerebral anatomy and can be superimposed directly on the real patient. This technology has the potential to improve anatomic visualization, surgical planning, and resident training, and therefore serve as a valuable mean to rehearse and plan the important steps of the surgery.^2-4^

The implementation of MxR has been promoted with success in different subfields of neurosurgery.^5-7^ Indeed, the use of 3D interactive holograms superimposable to the real world augments the anatomic perception and helps intraoperative orientation.^2^ Furthermore, 3D holograms allow not only experienced neurosurgeons but also, and especially, residents to rehearse the surgical steps, plan patient positioning and the surgical trajectories, and gain stronger surgical confidence.^3,4^ Nonetheless, the use of MxR in the routine neurosurgical practice is still not as diffuse as expected. In the past, a major limitation to the widespread use of MxR have been represented by the restrictions in computational and graphics processing ability.^8-10^ Nowadays, a precise evaluation of the cost-effectiveness of MxR is still difficult, given the ongoing experimental nature of the technology.^4^

Segmentation is the fundamental step to produce high-quality 3D holograms. It can be divided into 3 groups: manual, semiautomatic, and automatic, the last group being potentially faster than the other 2 options because it does not require any user input. To make the use of a MxR system adaptable to the routine surgical planning and the use of neurosurgeons at all levels of experience, the workflow must be mostly, if not fully, automatic and require the least amount of steps to reach the goal. The incorporation of algorithms for the automatic recognition of anatomic structures, such as skin, brain, ventricles, and tumors, in a cloud environment facilitates the workflow and eliminates the need of inputs from the users and of high-power local computers.

In the settings of digitalization of medicine and the growing drive toward 3D visualization of medical imaging, the interest to apply MxR in the form of 3D interactive holograms for surgical planning, case rehearsal, and training in neurosurgery is manifest and it is increasing. Nevertheless, this technology is still developing and there is a lack of studies focusing on its applicability in the clinical routine.

We present the first single-center experience gathered after routine implementation in 2022 of such a cloud-based MxR neurosurgical preparation system (Lumi, Augmedit bv) on 107 consecutive neurosurgical cases. This study endorses the applicability of MxR to routine neurosurgical planning, case rehearsal, and training.

## METHODS

Swiss ethics approved this study (BASEC nr Reg-2022-01015). All patients provided consent for imaging data use for this study under general KEK PB_2017_00093/NCT01628406.

The intraoperative use of the MxR system still represents an off-label use.

### Prospective Patients' Cohort

All patients who underwent an elective neurosurgical procedure in our Institution from January 2022 to December 2022 and for whom a hologram was prepared on request of the primary surgeon were included. Simple demographic data, ie, age at the time of surgery, and data regarding the surgical procedures were collected. Surgical preparation time was measured, defined as the time from the end of anesthesia induction to the beginning of the surgical procedure. This parameter was also measured in a historical cohort of cases performed in 2021. The 2021's cases were matched according to the indication of the surgical procedure (elective), the type pf the neurosurgical pathology, and the primary operating neurosurgeons.

### Segmentation and Holographic Rendering

Automatic segmentation of ventricles, major dural sinuses, brain, skull, skin, and eventual tumor was performed by means of an expanding meshes algorithm that was previously described and validated (Disior).^9,11^ The algorithm used 3D adaptive spheres that capture the radiological boundaries of different tissues. For each source imaging scan, the algorithm was specifically tailored to the patient by preprocessing using region, threshold, and histogram-based segmentation methods (9). This algorithm was run in a cloud environment that provided direct MxR output (Lumi, Augmedit bv). If subsequent structures were requested or automatic output was insufficient, semiautomatic contrast-based and full manual slice-by-slice segmentation of anatomic structures of interest was performed on 3D Slicer (3D Slicer image computing platform|3D Slicer) by the first author. The manual segmentations were converted to 3D surface models and exported as stereolithography files into the cloud environment. The resulting hologram was visualized using the HoloLens 2 (Microsoft Corporation) and the view of the primary user was directly streamed to a side screen in the operation room using Miracast (Microsoft Corporation).

Therefore, the novel MxR system used for holographic rendering and 3D visualization consisted primarily of a Conformité Européenne-certified cloud environment, which provided a direct MxR output by means of a validated expanding meshes algorithm. A further component was the associated application for MxR glasses, which represented the main tool for 3D visualization of the hologram.

**FIGURE 1.**
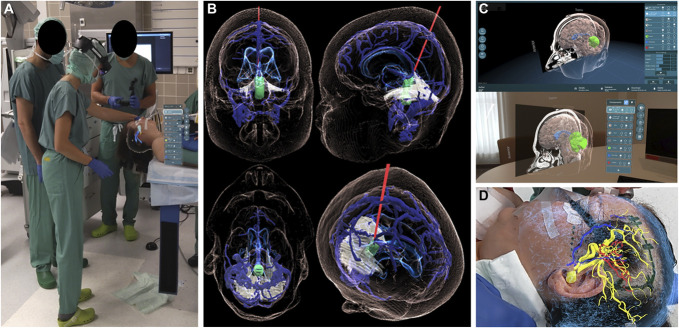
**A**, Matching of the hologram with the patient's head in the operating room and **B**–**D**, exemplifying preoperative and intraoperative hologram's use.

In the application Lumi for Microsoft Hololens 2, the surgeon could subsequently review the position the patient, place an incision, place a trajectory, and perform a trepanation with hologram manually placed next or onto the patient head (Figure 1, Video).

### Applicability of the System

**FIGURE 2.**
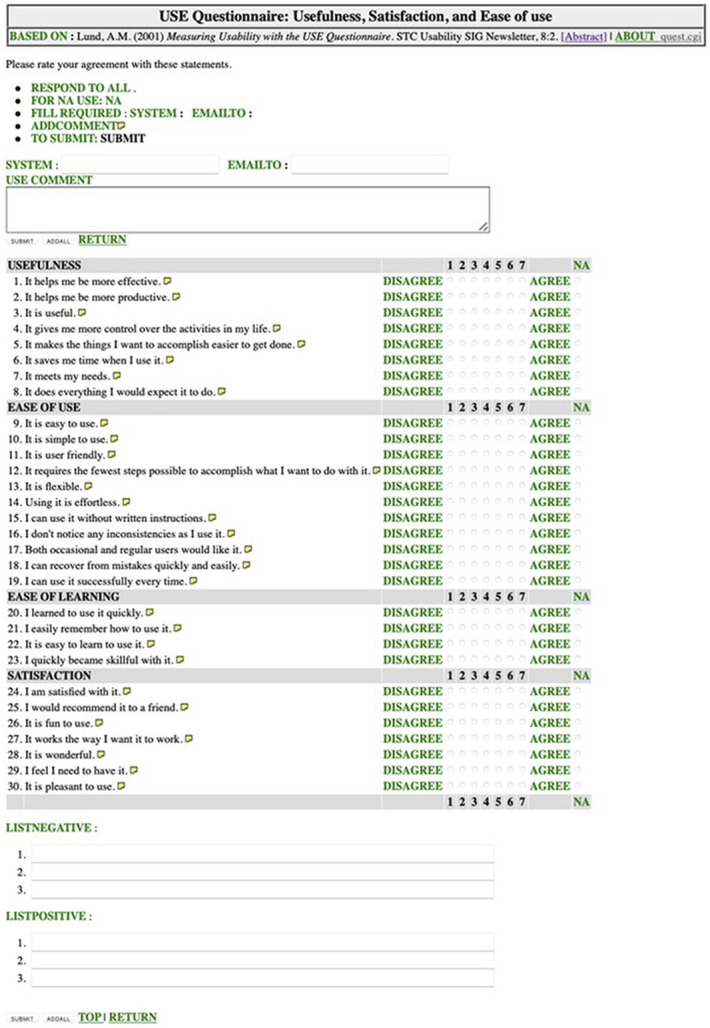
USE questionnaire. USE, Usefulness, Satisfaction and Ease of use.

Seven board-qualified neurosurgeons with a minimum of 3 months of experience with the system were questioned. Each of them listed up to 3 positive and 3 negative aspects of the system and filled out a Usefulness, Satisfaction and Ease of use (USE) questionnaire, composed of 30 Likert scale questions grouped into 4 categories: usefulness, ease of use, ease of learning, and satisfaction^12^ (Figure 2). Each neurosurgeon filled the questionnaire approximately after 3 months of experience with the system. For the purpose of the present analysis, an adequate usability was defined as a mean score of minimum 5 in each of the 4 tested categories.^13,14^

### Statistics

Descriptive statistics (medians and percentage for categorical variables) of the holograms' preparation time, the surgical preparation time, and the USE questionnaire scores were computed to provide an overview. The comparison between mean surgical preparation time with and without MxR was performed computing the 95% CI for the difference between 2 means.

## RESULTS

### Patients

In total, 107 holograms were prepared for 105 patients (Table 1).

**TABLE 1.** Patient Categories

Indication	Neoplasms	Vascular	CEA
Total number	63	27	17
Mean age at surgery, y	57 ± 15	57 ± 12	72 ± 10
Infratentorial	11 (11/63, 17%)	1 (1/27, 4%)	NA
Most represented pathologies	Meningiomas (32/63, 51%) Gliomas (14/63, 22%) Metastases (4/63, 6%) Pituitary adenomas (2/63, 3%) Others (11/63, 1%)	IA (17/27, 63%) Bypass (6/27, 22%) AVM (4/27, 15%)	NA
CEA laterality			Right 10/17 (59%) Left 7/17 (41%)

AVM, arteriovenous malformation; CEA, carotid endarterectomy; IA, intracranial aneurysm; NA, nonapplicable.

### Source Imaging

The most represented source imaging modality in the overall cohort was MRI (90/107 cases, 84%), specifically: T1 contrast-enhanced MRI sequences, which were used to segment skin, brain venous structures, and some tumors, and MR angiographies with time-of-flight sequences, which were used to segment the intracranial and extracranial arterial vessels. CT-angiography studies (11/107 cases, 10%) followed in frequency and were used exclusively for carotid endarterectomy (CEA) cases. The holograms of 3 of 107 cases (2.8%) were realized segmenting structures from both imaging sources.

### Hologram Preparation

Mean time of hologram preparation, from the start of segmentation to complete holographic rendering, was 39.4 ± 20.4 minutes for the overall cohort. Per surgical indication, mean preparation time was 52 ± 21 minutes for neoplasms, 28.3 ± 12 minutes for vascular cases, and 25 ± 6 minutes for CEAs. Automatic segmentation of skin and brain was achieved in a total of 95 cases (95/107, 89%), of the lateral ventricles in 59 cases (59/107, 55%), of the biggest venous sinuses in 48 cases (48/107, 45%), and of the tumors in 31 cases (31/107, 29%). A semiautomatic skull segmentation was added in 76 cases (76/107, 71%) and a manually segmented anatomic structure was added in 75 cases (75/107, 70%). Most frequent manually segmented and added structures were the superficial temporal artery in the vascular subgroup (5/27, 19%), complex tumors and specific venous structures in the oncologic subgroup (respectively: 33/63, 52%, and 24/63, 38%), and the carotid artery complex (common carotid artery, internal carotid artery, and external carotid artery) in the CEA subgroup (10/17, 59%).

### Surgical Preparation

Mean surgical preparation time was 48 ± 17 minutes in the whole cohort of MxR cases compared with 52 ± 17 minutes in the historical cohort (mean difference 4, 95% CI 1.7527-9.7527).

In 3 of 107 cases (2.8%), the preoperative 3D visualization drove the primary operators to modify the foreseen surgical approach. In the first case, an M1 aneurysm clipping, a planned laterosupraorbital craniotomy was changed into a pterional craniotomy to gain more oversight over a M2 segment. In the second case, again a middle cerebral artery-aneurysm clipping, the head of the patient was rotated more to gain a better exposure of the proximal sylvian fissure. In the third case, a supracerebellar infratentorial approach was changed into an interhemispheric transtentorial approach after hologram review to operate a lesion extending from the IV ventricle to the lamina quadrigemina in close relation with the basal cerebral veins to better protect the latter.

### Usability Results

A mean score of 5 out of 7 was documented for usefulness, 5 for ease of use, 6 for ease of learning, and 5 for satisfaction, showing good usability in all 4 USE questionnaire categories. Four of the 7 (57%) interviewed neurosurgeons gave a mean score equal or above 5 to all 4 categories. Among the most negative aspects of the system listed by the tested neurosurgeons, the most recurrent was the nonideal manual mechanism to match the hologram with the head of the patients (5/7, 71%), which may be imprecise and need adjustments, therefore requiring time. This comment was followed in frequency by the dependence of the system on a good internet connection (2/7, 29%). The most frequent greatest positive aspect of the system was the better understanding of the anatomy and the pathology, thanks to the holographic 3D visualization (5/7, 71%). The second most frequent (3/7, 43%) positive aspect for the 7 neurosurgeons was the ease of use of the system. Table 2 provides a detailed overview of the results of the USE questionnaires.

**TABLE 2.** Results of the USE Questionnaires

Experience	Usefulness								Ease of use											Learning				Satisfaction							Mean score per surgeon (SD)
	1	2	3	4	5	6	7	8	9	10	11	12	13	14	15	16	17	18	19	20	21	22	23	24	25	26	27	28	29	30	
Junior attending	6	6	6	6	6	6	6	6	6	6	6	5	5	5	5	5	6	5	5	6	6	6	6	6	6	6	6	6	6	6	5 (0.6)
Chief resident	6	6	6	6	6	6	6	6	6	6	6	5	5	5	6	5	6	7	6	7	7	7	7	6	7	7	7	6	6	6	6 (0.3)
Specialist attending	4	4	5	5	4	5	6	6	5	5	6	5	5	5	5	5	5	4	4	7	7	7	7	5	7	7	6	5	5	5	6 (0.5)
Chief	7	5	5	4	5	5	3	3	4	4	4	4	4	4	5	5	6	5	5	6	6	6	6	4	7	7	3	5	5	6	6 (0.9)
Senior attending	7	4	7	5	5	2	4	3	4	4	4	6	4	3	5	4	7	4	3	5	6	5	5	5	6	6	4	4	4	6	5 (0.6)
Senior attending	7	4	7	5	5	2	4	3	4	4	4	6	4	3	5	4	7	4	3	5	6	5	5	5	6	6	4	4	4	6	5 (0.3)
Specialist attending	3	3	3	5	4	1	2	2	7	7	7	3	4	4	4	7	5	5	5	5	4	5	6	6	6	5	5	NA	2	7	5 (1)
Specialist attending	3	2	4	3	2	1	4	3	4	3	4	4	2	4	2	3	2	1	5	4	5	4	5	4	3	5	4	4	4	5	4 (0.7)
Mean score per category (SD)	5 (1.6)								5 (1.2)											6 (1)				5 (1.2)							

USE, Usefulness, Satisfaction and Ease of use.

## DISCUSSION

With the upcoming availability of Conformité Européenne-certified MxR systems, the routine implementation of MxR may have the potential to improve intraoperative efficiency and the outcomes of surgery. The current prospective collection of cases is, to our knowledge, the most extensive to study a Conformité Européenne-approved MxR system for neurosurgical preparation. As an initial step, we focused on usability, user feedback, and influence on surgical preparation.

Other groups have published on the implementation of AR systems in the workflow of specific neurosurgical procedures and pathologies with the aim of evaluating usability and added value. Moon et al used MxR for multidisciplinary awake craniotomy planning in 10 consecutive cases and endorsed the use of MxR showing acceptable usability and benefit for surgical rehearsal.^15^ The group of Sugiyama et al published in 2020 their experience on the use of an immersive virtual reality (VR) system implemented for the presurgical discussion and preparation of 18 cerebrovascular cases. They endorsed the value of VR to improve patient-specific anatomic understanding, surgical strategy in complex cases, and training for less experienced neurosurgeons.^16^ Recently, Zawy Alsofy et al published the experience gathered with surgical planning using VR in different neurosurgical subfields, including neuro-oncology, vascular, and spine.^17-21^ Their results supported the conclusion that VR only moderately affected conventional patient positioning but it did help better detection of pathology-specific anatomic relationships. The use of AR has also been extended to intraoperative visualization of major anatomic landmarks, as documented by Bernard et al,^22^ to improve periventricular structure preservation in 13 cases of tumor removal.

### Impact of MxR on the Surgical Routine

The present analysis shows that the application of the tested MxR system in the operating room during the nonsterile preparation phase does not increase the surgical preparation time. Time required by the above-mentioned process may have indeed had a negative impact on the surgical preparation timing, ie, increasing it, thus affecting also the total surgical timing. This aspect may implicate that a more precise preoperative 3D visualization could not only provide a better anatomic understanding but also the development of a more definitive preoperative plan (surgical approach and patient positioning namely), thus improving efficiency of surgical planning. Furthermore, the increasing frequency of application of this technology over the course of the year demonstrated the positive perception of the neurosurgeons and established its role as a beneficial adjunct of surgical planning.

Indeed, the most significant contribution of the tested MxR workflow was seen in the 3 cases, for which the perioperative 3D study helped optimizing surgical approach and patient positioning, thus the intraoperative trajectory. Three cases represent a relative small percentage compared with the total number of procedures included in this study; nevertheless, the 3D interactive holograms added a better specific anatomic understanding and provided experienced neurosurgeons with what they considered an improved surgical planning and intraoperative strategy. With further and more extensive application of this technology, it may have the potential to objectively influence other procedures and therefore improve surgical and functional outcomes in the future.

### Users' Experience and Satisfaction

The analysis of the data gathered through the USE questionnaires documented overall a good usability of the system and users' satisfaction. Currently, there is no standardized usability assessment for AR systems. Nonetheless, the goals of the present analysis' evaluation method are aligned to the ones of the previous studies, aiming mainly to evaluate usefulness, ease of use and learning, confidence of the users with system, and users' satisfaction.^15^ A further recurrent aspect was the identification of an improved anatomic understanding as the most relevant benefit of the application of MxR to surgical planning and preparation.^5-7,17^ Indeed, the younger colleagues interviewed provided overall the most positive appraisal of the system and its perioperative application. The resulting implication may be that the greatest value of MxR implemented for surgical planning and rehearsal is mostly evident during neurosurgical training and for less experienced neurosurgeons. A bigger cohort of interviewed neurosurgeons with different levels of experience would be necessary to confirm this theory.

### Limitations of the System

Working with the current MxR system still included some drawbacks. First, the degree of manual segmentation necessary to produce high-quality holograms is still quite high in the present cohort, with at least 1 manually segmented anatomic structure in more than 50% of the included cases in all 3 categories (ie,: vascular, oncologic, and CEAs). Ideally, this should be reduced by improving the current algorithms and developing new ones. Machine learning could be used for both purposes. Furthermore, the algorithm for automatic segmentation of skin, brain, ventricles, and tumors used in the present analysis (Disior) works with indirect training of data and it is optimized for contrast-enhanced tumors with a minimum volume of 5 cubed-cm on T1-weighted imaging. These features require a larger data set to improve the sensitivity of the algorithm for smaller tumors.^11^

Another limitation is intrinsic to the nature of the quality of the imaging. In fact, segmentation of vessels with a diameter below 1 mm and the smallest anatomic structures, such as cranial nerves other than the ophthalmic and the trigeminus, was not possible. Indeed, the algorithm for automatic segmentation used in this study is not designed to recognize arterial vascular structures. This is not solely a limitation of this study. Given the intrinsic anatomic difficulty in segmenting small intracranial arteries, gold standard algorithms to provide automatic and successful segmentation of cerebral are still a work in progress.

Further limitations of the system have emerged after testing neurosurgeons using it for more than 3 months; in fact, the matching of the hologram with the head of the patient is currently manual, thus requiring an input from the user and care to make it as precise as possible. Another relevant limitation expressed by the neurosurgeons is the influence of a good internet connection to the performance of the MxR system.

### Limitations of the Study

This study has several limitations. First, all the 107 included cases have been selected from a single neurosurgical tertiary referral center with a relevant caseload, expertise, and high adaptation to new technological developments, leading to a selection bias. Furthermore, all included cases represent surgeries performed in an elective regime and no emergency procedures have been included in the analysis. Another limitation pertains to the tested population of neurosurgeons. In fact, only one chief resident had gained enough experience with the system and no younger residents were included in the analysis. Furthermore, in this initial phase of implementation of the MxR system, any consideration about the improvement of the surgical planning after preoperative hologram's study is still subjective to the specific neurosurgeon using the technology. Before making objective conclusions, an analysis of more neurosurgical cases including the surgical outcomes will be necessary.

### Potential Improvements and Future Perspectives

The first and most relevant improvement for the tested MxR system would be the development of machine learning algorithms not only designed to recognize cerebral tumors, but also vascular pathologies and fine anatomic structures (ie, cranial nerves). As far as surgery rehearsal is concerned, the MxR system used did not provide the users with haptic feedback, nor did it allow the simulation of dural opening/closing or arachnoid dissection. These aspects should ideally be integrated to the system in the future.

The lack of more specific data gathered on the use of the hologram, for instance, the total time of use of the hologram in the OR, represents a sensible goal for a subsequent study to document the impact of MxR on the OR workflow and multidisciplinary team.

Future research should focus on improving the realism of the anatomic reconstruction of the system, empowering the users to rehearse all steps of the surgical procedures. With further technological development, one for all the integration of hemodynamic information, for instance, routine implementation of MxR will have indeed the potential to make a critical difference in all subfields of neurosurgery. Indeed, further attention should be given to automatic face hologram matching and to improve the performance of the system with nonoptimal internet connection.

## CONCLUSION

To our knowledge, this study analyzes the biggest single-center series of neurosurgical cases prepared with the use of 3D interactive holograms, documenting the impact of MxR's implementation in the neurosurgical routine. The perioperative use of 3D holograms improved direct anatomic visualization while not significantly increasing intraoperative surgical preparation time. Usability of the system was adequate. Further technological development is necessary to improve the automatic algorithms, reduce the hologram preparation time by circumventing manual and semiautomatic segmentation, make the holograms more realistic, and reduce even further the steps required to use the system. Future studies should focus on quantifying the benefits in teaching, training, and the impact on surgical and functional outcomes.

## COMMENTS

This report by Colombo et al is a timely addition to the growing literature surrounding the implementation of augmented or mixed reality in the neurosurgical operating room. Frameless stereotaxy has revolutionized cranial surgery and has been universally adopted; augmented reality almost certainly represents the next generation of this technology. The primary hurdle to such implementation, aside from cost, is user familiarity and acceptance. Therefore, the report of this technology in over 100 cases, with no increase in preoperative planning times, should encourage readers that this technology can be seamlessly added to the workflow of a modern operating room.

Nonetheless, this case series illustrates how far augmented reality still must go to become clinically useful. In only 3 of 107 cases was the operative plan altered by the addition of this technology; 2 of these, aneurysm clippings, would be performed by most surgeons without the use of stereotactic guidance in the first place, while the third, a tectal-region mass, was altered from an infratentorial to a transtentorial approach. This is a significant change that would realistically require repositioning of the patient, and in our experience, such a change would need to be made preoperatively by detailed review of MRI imaging. We feel that an experienced surgeon planning such an approach is unlikely to be influenced by the low-resolution 3D reconstructions demonstrated in the accompanying figures, while, on the other hand, these images have not yet reached the level of precision and reliability to assist the inexperienced surgeon either. Therefore, these tools are currently most useful in teaching residents the crucial internal three-dimensional understanding of cranial anatomy that previously could only be gained from repetitive surgical experience. But as this report implicitly acknowledges, their usefulness ceases once incision is made.

**Peter Theiss and Ali Alaraj**

*Chicago, Illinois, USA*
